# Novel 3D-Printed Replica Plate Device Ensures High-Throughput Antibacterial Screening of Halophilic Bacteria

**DOI:** 10.3390/md23080295

**Published:** 2025-07-23

**Authors:** Kaloyan Berberov, Nikolina Atanasova, Nikolay Krumov, Boryana Yakimova, Irina Lazarkevich, Stephan Engibarov, Tsvetozara Damyanova, Ivanka Boyadzhieva, Lyudmila Kabaivanova

**Affiliations:** 1 Department of General Microbiology, The Stephan Angeloff Institute of Microbiology, Bulgarian Academy of Sciences, 1113 Sofia, Bulgaria; nikolina@microbio.bas.bg (N.A.); krumov.1993@gmail.com (N.K.); irinalazarkevich@abv.bg (I.L.); stefan_engibarov@abv.bg (S.E.); tsvetozaradamianova@gmail.com (T.D.); petrovaim@abv.bg (I.B.); 2 Laboratory of Chemistry and Biophysics of Proteins and Enzymes, Institute of Organic Chemistry with Centre of Phytochemistry, Bulgarian Academy of Sciences, 1113 Sofia, Bulgaria; boryana.yakimova@orgchm.bas.bg; 3 Institute of Neurobiology, Bulgarian Academy of Sciences, 1113 Sofia, Bulgaria; 4 Department of Biotechnology, The Stephan Angeloff Institute of Microbiology, Bulgarian Academy of Sciences, 1113 Sofia, Bulgaria; lkabaivanova@microbio.bas.bg

**Keywords:** 3D-printed, agar overlay, antimicrobial compounds, brine, halophiles, halophilic bacteria, high-throughput, replica plate, saltern, *Virgibacillus*

## Abstract

Antibiotic resistance is one of the most significant public health issues today. As a consequence, there is an urgent need for novel classes of antibiotics. This necessitates the development of highly efficient screening methods for the rapid identification of antibiotic-producing bacteria. Here, we describe a new method for high-throughput screening of antimicrobial compounds (AMC) producing halophilic bacteria. Our methodology used a newly designed 3D-printed Petri plate replicator used for drop deposition and colony replication. We employed this device in combination with a modified agar overlay assay to screen more than 7400 bacterial colonies. A total of 54 potential AMC producers were discovered at a success rate of 0.7%. Although 40% of them lost their antibacterial activity during the secondary screening, 22 strains retained inhibitory activity and were able to suppress the growth of one or more safe relatives of the ESKAPE group pathogens. The ethyl acetate extract from the most potent strain, *Virgibacillus salarius* POTR191, demonstrated moderate antibacterial activity against *Enterococcus faecalis*, *Acinetobacter baumanii*, and *Staphylococcus epidermidis* with minimal inhibitory concentrations of 128 μg/mL, 128 μg/mL, and 512 μg/mL, respectively. We propose that our replica plate assay could be used for target-based antimicrobial screening of various extremophilic bacteria.

## 1. Introduction

Almost a century after the discovery of the first antibiotic, penicillin, and five decades after the discovery of the last antibiotic from a new class, daptomycin, human society faces a new global health problem—exponentially increasing antibiotic resistance (ABR) among bacterial pathogens [1,2]. The World Health Organization (WHO) identifies ABR as one of the top ten global public health issues of the 21st century [3]. Approximately 33,000 deaths are attributed annually to ABR in the European Union [4], and 700,000 deaths are registered on a global basis [5]. According to a report by the UK government from 2014, if no new class of antibiotics is discovered by 2050, the total number of deaths caused by drug-resistant pathogens could reach 10 million annually [6].

However, traditional platforms for antibiotic discovery have largely been abandoned due to declining success and reduced funding. Systematic screening of actinomycete strains or other common soil microorganisms is now largely neglected due to the repeated rediscovery of a limited number of the same producers and compounds that have already been well characterized [7]. This highlights the need to develop new antibiotic discovery strategies [8]. As a result, we now observe a redirection of research interest towards screening for AMC in so far uncultured microbial species or poorly explored ecological niches which has achieved some promising results [9,10,11]. Unusual microbial habitats such as fresh water and marine sediments, coral reefs, extreme soils, deserts, caves, and endosymbionts are now largely screened for producers of AMC inhibiting the growth of human pathogens [12]. Extremophilic bacteria are a notable example for surviving and thriving in diverse harsh (extreme) physicochemical environmental conditions and have been identified as untapped source of secondary compounds including antimicrobials [13]. In particular, halophilic bacteria living in high-salt environments (salt mines, solar salterns, saline soil, saline sediments, and saline lakes) are now beginning to be recognized as a novel natural source of antimicrobial compounds. A diverse range of halophilic bacteria, fungi, and algae have been extensively screened for AMC production [14].

Nevertheless, the screening process requires high-throughput methods allowing large-scale isolation of target AMC producers. Highly advanced techniques such as microfluidics and mass spectrometry have been developed to ensure high-throughput analysis of large bacterial libraries but require specialized equipment that can be expensive and inaccessible [15,16]. Genome mining for biosynthetic gene clusters (BGCs) encoding AMC has also been applied to environmental metagenomic libraries, but these BGCs are often silent and downregulated and, therefore, not expressed under laboratory conditions [17]. On the other hand, the culture-dependent isolation and direct screening of bacterial cells or colonies has proven to differentiate those bacteria that can produce AMC in vitro [18,19]. The co-culturing of multiple strains at once further stimulates AMC production [20]. Consequently, agar-based methods (agar well diffusion, agar disk diffusion, agar overlay, and agar plug assays) are still usually used at the initial stage of the screening as they provide reliable, simple, and low-cost detection. They rely on the tridimensional diffusion of the produced AMC through the agar layer(s), which leads to formation of inhibitory zones in the lawn with test pathogen. However, these methods are laborious, as they require initial creation of large collections of pure strains that have to be screened individually.

Several attempts have been made to scale down this process by developing a pipeline for screening multiple isolates at once. Robust strategies have been employed by dual-sided agar assay with specially made for the purpose double-faced rectangular Petri plates (*Janus* plates) [18] or 96-well plates (DAPA plates) [15]. In this assay, the producer and indicator strain are grown on the two opposite sides of the agar poured in the specially manufactured plates. To circumvent the use of custom-designed plates, some authors used a direct agar overlay of mixed culture colonies grown on agar plates [21]. More convenient alternative of this assay is the grid-like spot inoculation of multiple producer strains at once, which after proper incubation are overlayed with top semi-solid agar previously inoculated with indicator pathogen [22,23]. The grid-like inoculation of the producer strains can be made less labor-intensive and less time-consuming by using a Petri plate replicator, which ensures highly parallelized large-scale screening [24,25,26].

The replica plate assay was first developed by Lederberg and Lederberg (1952) [27] using velvet or velveteen fabric to transfer grown colonies on new agar plates while retaining their location and size. To avoid the frequently observed colony smearing and uneven inoculum deposition [28], several different models of Petri plate replicators have been proposed. They were generally made with stainless steel nails [29] or stainless-steel pins [30,31,32] attached to a common base. Nowadays, these replicators made in-house are no longer used in favor of expensive commercially made ones designed to fit half or whole standard 96-well plate and then transfer inoculum material to Petri plates. While these replicators have been employed in most of the studies mentioned above [18,20,24,33], their primary limitation lies in the necessity to first obtain pure cultures of the producer strains. Liquid cultures of these pure strains are then assembled as libraries in 96-well plates, replicated on agar plates and screened for antagonistic activity.

To circumvent this laborious and time-consuming stage of the conventional primary screening procedure, we developed a novel 3D-printed Petri plate replicator, which can be made by anyone with access to 3D printer. This device enables simultaneous isolation and antibacterial screening of multiple single-cell-derived colonies. To validate the efficacy of the method, we applied it to a library of over 7400 halophilic bacterial colonies isolated from three hypersaline environments in Bulgaria. Those colonies that exhibited inhibitory activity were readily retrieved for further characterization of their antimicrobial potential against safe relatives of the ESKAPE group pathogens. This culture-based approach enabled the inoculation of several hundred colonies a day, thereby substantially accelerating high-throughput screening and enhancing the likelihood of isolating AMC producers. We believe that our study will further stimulate the search for novel rapid and large-scale screening methods that will facilitate antibiotic discovery.

## 2. Results

### 2.1. Physicochemical Parameters of the Samples

Some physicochemical parameters of the samples collected from Provadia salt deposit, Burgas salterns, and Pomorie salterns (Bulgaria) are summarized in Table 1. The saline soil samples from the Provadia salt deposit had an overall slightly alkaline pH. The pH of the samples from the South zone of the deposit was 8.2, while those from the East zone had a pH of 8.2–8.4. The samples taken from the crystallizer pond of the deposit also had a slightly alkaline pH of 8.0. The soil temperature (34 °C) was uniform among the samples. According to the soil salinity classification of Richards (1954) [34], the samples from the East zone were very slightly saline (2.93 ± 0.04 dS/m) while the samples from the South zone were slightly saline (7.26 ± 0.24 dS/m). The soil samples from the crystallizer pond were strongly saline (20.30 ± 0.61 dS/m).

The temperature of the brine above the saline mud from the Burgas salterns was higher and in the range of 35.4–38.0 °C. The samples from the feeding pond had a lower salinity of 7.3–9.8% while those from the final crystallizer ponds had a higher salinity of 20.9–29.0%. The pH was 8.0–8.2 for the feeding ponds and 7.0–7.5 for the crystallizer ponds.

The same correlation was observed for the samples from the Pomorie salterns. The samples from the feeding ponds had lower salinity (10.0–13.8%) and slightly higher pH (7.9–8.2). The crystallizer pond samples were characterized by a high salinity of 27.0% and a pH of 7.8. Pomorie Lake had the lowest salinity of 5.9% and an alkaline pH of 9.0. The temperature of the samples was fairly uniform (between 25.8 °C and 28.0 °C). More detailed data on the physicochemical properties of all individual samples can be seen in Table S1.

### 2.2. Culturable Bacteria Cell Count

A critical step in the primary screening developed in our study was to enumerate the culturable bacterial cells in the samples. For that reason, we conducted a bacterial cell count by the simple and rapid single plate-serial dilution spotting assay [35]. The concentration of the culturable bacteria was enumerated by spot inoculation and cultivation on the optimal culture medium for the respective samples (see next section). The samples from the Provadia salt deposit and the Pomorie salterns were cultured on HM medium [36], while those from the Burgas salterns were cultured on Rodriguez-Valera medium [37].

The bacterial abundance in the samples from the two zones of the Provadia salt deposit was comparable—3.08 ± 4.56 × 10^4^ CFU/g for the South zone and 1.97 ± 2.96 × 10^4^ CFU/g for the East zone. The crystallizer pond was characterized by a lower cell concentration of 0.37 ± 0.08 × 10^4^ CFU/g. The bacterial cell count of the saline mud samples from the Burgas salterns did not differ significantly between the feeding and crystallizer ponds and was in the range of 1.66−8.08 × 10^5^ CFU/g. The bacterial abundance in the brine samples was lower (0.01–4.55 × 10^4^ CFU/mL) compared to the saline mud samples, with the exception of one brine sample with 80.00 ± 17.32 × 10^5^ CFU/mL. All values were 10-fold higher than those obtained from the other salterns near Pomorie town. In that hypersaline environment, the cell concentrations varied from 3.15 ± 2.49 × 10^4^ CFU/g to 8.49 ± 1.04 × 10^4^ CFU/g for the saline mud samples. The observed bacterial abundance of the brine samples was higher (9.10–11.20 × 10^4^) compared to the mud samples.

### 2.3. Optimized Bacterial Cell Recovery

A pivotal step in the isolation of bacteria from environmental samples is the proper detachment and/or desorption of the bacterial cells from the complex sample matrix. The optimal cell extraction provides a higher cell yield, which leads to a more profound isolation of a wider diversity of strains presented in the sample. Usually, this procedure involves a combination of chemical and physical treatments of the samples to provide sufficient cell recovery [38].

**Figure 1:**
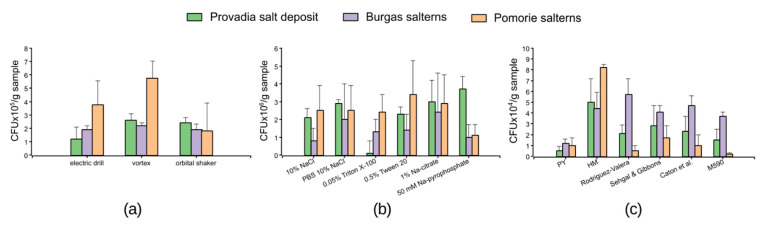
Effect of (**a**) different physical dispersion regimes, (**b**) chemical dispersants, and (**c**) culture media on the recovery of bacterial cells from the samples as determined by the final CFU per g of sample.

The three regimes of physical dispersion of the samples did not significantly influence the final CFU/g from the samples of the Provadia salt deposit and the Burgas salterns. The cell recovery was in the range of 1.2–2.6 × 10^5^ CFU/g. More evident differences were observed for the saline mud samples from Pomorie salterns. However, for all the samples, the highest cell count was obtained when vortexing for 3 × 1 min was used (Figure 1a). The different chemical dispersants used for sample suspension evidently influenced the cell recovery (Figure 1b). The optimal dispersant for the saline soil samples from Provadia was found to be 50 mM sodium pyrophosphate (3.7 ± 0.7 × 10^6^ CFU/g). The best results (2.4 ± 2.2 × 10^6^ CFU/g) were obtained with 1% sodium citrate as a dispersant of the saline mud samples from Burgas salterns. On the other hand, the use of 0.5% Tween 20 led to the highest cell count (3.4 ± 1.9 × 10^6^ CFU/g) from the saline mud samples collected from Pomorie salterns. The type of culture medium had the strongest influence on the cell recovery, as shown in Figure 1c. Six culture media with different quantitative and qualitative compositions of salts and organic compounds were used. Overall, the peptone yeast extract medium (PY) used in previous studies on the biodiversity of halophilic bacteria from Burgas and Pomorie salterns [39] gave the lowest cell counts of 0.5–1.2 × 10^4^ CFU/g (with the exception of the Pomorie salterns samples, where M590 had the lowest cell yield). Higher cell counts (4.4–8.2 × 10^4^ CFU/g) were obtained using HM medium, which was the optimal isolation medium for the samples from Provadia salt deposit (5 ± 2.2 × 10^4^ CFU/g) and Pomorie salterns (8.2 ± 0.3 × 10^4^ CFU/g). Rodriguez-Valera medium was recognized as more suitable for the saline mud samples from Burgas salterns (5.7 ± 1.5 × 10^4^ CFU/g).

### 2.4. Optimal Design of the 3D-Printed Petri Plate Replicator and Method Validation

To find the most suitable pin diameter, we made three different Petri plate replicators with 1 mm, 2 mm, and 3 mm pin diameters (Table 2). The 3 mm pins had a 100% success rate of colony growth after drop deposition, but they deposited twice the desired volume (2.2 μL) of sample suspension, which led to the growth of two colonies from a pin on average. This was not suitable for the subsequent primary screening and picking of pure cultures from the agar plates. The 1 mm and 2 mm pins had slightly lower success in cell deposition, but resulted in the inoculation of only one cell per pin. Because of the higher success rate (92%) and proper drop volume of 1 μL, the Petri plate replicator with 2 mm pins was chosen as the most suitable design.

The use of aseptic techniques and sterility maintenance is crucial during microbiological experiments. Because neither PLA nor PETG is heat-stable, autoclaving the replicator was not applicable. On the other hand, sterilization by autoclaving at every Petri plate replication would make the procedure time-consuming. Therefore, to avoid cross-contamination between two subsequent Petri plate replications, we applied different chemical sterilization procedures to the replicator before drop deposition and colony replication. The shorter periods of 70% ethanol immersion (from 5 s to 20 s) were not sufficient for proper sterilization of the plastic pins. Occasionally, 1–2 colonies were seen on the agar plates touched with the sterilized plastic pin replicator. After 30 s and 60 s immersion in 70% ethanol, no bacterial growth was observed. The use of a Petri plate replicator with plastic pins to replicate grown colonies was not suitable because of the repetitive cross-contamination observed between two subsequent replications. Proper sterilization was not achieved even with 5 min immersion in 30% H_2_O_2_ combined with 5 min immersion in 70% ethanol. As a result, for the colony replicator, we employed a different strategy by substituting the plastic pins with metal ones. With this new design, a simple 30 s immersion in 70% ethanol and one-time flame sterilization was sufficient to achieve sterility of the metal pins. The success of sterilization was further increased by wiping the metal pins with sterile filter paper after they had been used to transfer colonies on the agar plates. After that, the pins were processed as described above.

**Figure 2:**
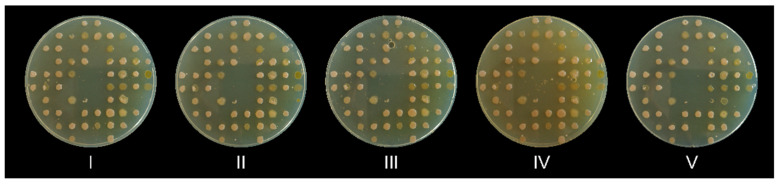
Five consecutive agar plates were replica plated with a single charging of the 3D-printed replicator. The plates were numbered with Roman numerals (I–V) in the order of their replication. Note that the small dots are air bubbles trapped in the agar, not a contamination.

Finally, after validating the replica plate process, we wanted to determine how many consecutive agar plates can be replicated. As could be seen in Figure 2, up to five agar plates can be inoculated with a single charging of the replicator’s pins without any evident loss of inoculum or contamination.

### 2.5. Replica Plate Guided Screening of Bacterial Isolates Library

**Figure 3:**
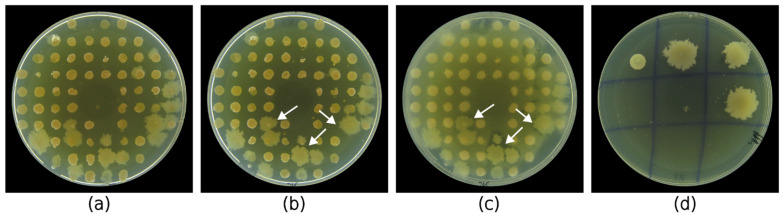
General workflow of the developed large-scale screening process. (**a**) The isolates were first isolated on HM or Rodriguez-Valera medium by drop deposition from the initial sample suspension, which was designated as the master plate. (**b**) Then, the 74 grown colonies were replica plated on two agar plates (replica plates). (**c**) After a modified agar overlay assay of the replica plates and incubation of the test pathogen, inhibitory zones could be seen around the active colonies (white arrows). (**d**) Finally, the bioactive colonies were retrieved from the master plate and isolated in pure cultures.

In this study, we aimed to develop a rapid and reliable method for large-scale antibacterial screening of bacterial isolates. To validate our method, we used it to screen halophilic bacteria isolated from 101 samples of saline soil, saline mud, and brine. Figure 3 shows the general workflow of the assay. After drop deposition of sample suspension, the grown colonies were replica plated with the 3D-printed replicator (Figure 3b) and screened for antagonistic activity by a modified agar overlay assay using *E. coli* and *S. epidermidis* as Gram-negative and Gram-positive test pathogens, respectively (Figure 3c). If we exclude the initial 72 h needed to isolate the screened colonies, the whole assay can be completed in four days.

**Figure 4:**
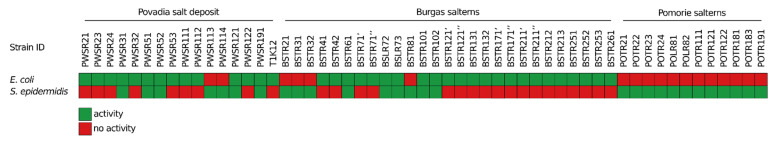
Antibacterial activity of the replica plated halophilic bacterial isolates during the primary screening against *E. coli* ATCC 25922 and *S. epidermidis* ATCC 12228.

This led to the simultaneous isolation and screening of more than 7400 colonies. From them, only 54 demonstrated antibacterial activity against one or both of the pathogens as determined by the inhibitory zones around the colonies devoid of pathogen growth (Figure 4). This represented a 0.7% successful hits of potential AMC-producing halophilic bacteria. More than half of the isolates (36 isolates; 67%) demonstrated antibacterial activity against *E. coli*. There were 27 (42%) isolates with antagonistic activity against *S. epidermidis*. A combined activity against two of the test pathogens was observed in only nine (17%) of the isolates. The largest number of AMC-producing bacteria (27 isolates) was isolated from Burgas salterns, while almost an equal number of producers were isolated from the samples of Provadia salt deposit (15 isolates) and Pomorie salterns (12 isolates). Most of the bioactive isolates were isolated from saline soil and saline mud samples. Isolates from only four samples of brine demonstrated antibacterial activity. The strains that demonstrated activity at the primary screening were retrieved from the respective master plate and isolated in pure culture (Figure 3d).

### 2.6. Antibacterial Activity Confirmation Against ESKAPE Group Safe Relatives

**Figure 5:**
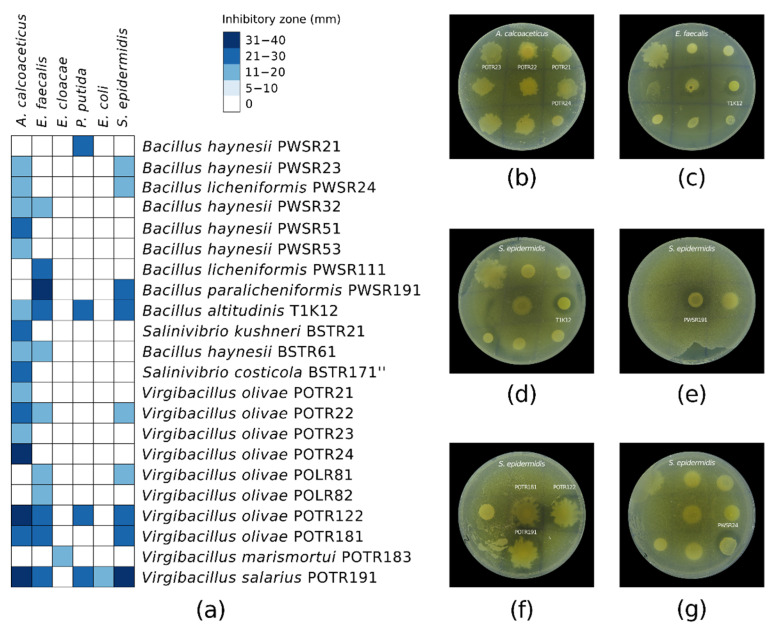
(**a**) Spectrum of antibacterial activity of the pure strains against a panel of ESKAPE group safe relative pathogens. The inhibitory zones of each isolate were measured in mm. Agar overlay assay showing the antibacterial activity of some of the pure strains against (**b**) *A. calcoaceticus* ATCC 23055; (**c**) *E. faecalis* ATCC 29212; (**d**–**g**) *S. epidermidis* ATCC 12228. Those strains from the primary screening that did not exhibit antibacterial activity at the secondary screening were omitted.

To confirm the antibacterial activity and to investigate the spectrum of inhibition of the active halophilic isolates, we subjected them to a secondary screening against a panel of six pathogens that are safe relatives of the ESKAPE group, as shown in Figure 5. A vast loss of activity against the initial test pathogens (*E. coli* and *S. epidermidis*) was observed. Furthermore, the activity of only 22 of the initial 54 isolates was confirmed. This represented 41% confirmed potential AMC producers from the initial isolates library detected at the primary screening. Only strain POTR191 remained active against *E. coli*, while the activity of a few strains (n = 9) was confirmed against *S. epidermidis*. All of the confirmed 22 isolates demonstrated inhibitory activity against at least one of the test pathogens. Most of the strains (77%) showed single or double antagonistic activity. A minor part of them (23%) were able to inhibit the growth of three or more test pathogens. The strains demonstrated poor antibacterial activity against *E. cloacae*, *P. putida*, and *E. coli*. On the other hand, most of the strains showed good growth inhibition of *A. calcoaceticus* (73%) and *E. faecalis* (50%). As determined by the agar overlay assay, the inhibitory zones varied from 12 mm to 40 mm. The semi-quantitative antibacterial activity of the strains was mainly expressed by 12–20 mm inhibitory zones.

The taxonomic affiliation of the confirmed active isolates and the reconstructed phylogenetic tree based on the 16S rRNA gene sequences are shown in Table S2 and Figure S1, respectively. The bioactive strains were mainly assigned to the genera *Virgibacillus* and *Bacillus*. Two of them were representatives of the genus *Salinivibrio*. The *Virgibacillus* sp. members demonstrated better activity inhibiting the growth of one to five of the test pathogens and producing wider inhibition zones (20–40 mm). *Bacillus* sp. manifested mostly single or double activity and narrower zones of 11–20 mm. One exception was *Bacillus altitudinis* T1K12, which showed antagonistic activity against four pathogens with inhibitory zones of 11–30 mm. The strains with the widest antibacterial spectrum were *B. altitudinis* T1K12, *Virgibacillus olivae* POTR122, *V. olivae* POTR181, and *Virgibacillus salarius* POTR191, with four, four, four, and five inhibited test pathogens, respectively.

### 2.7. Mass AMC Production in Liquid Media

**Figure 6:**
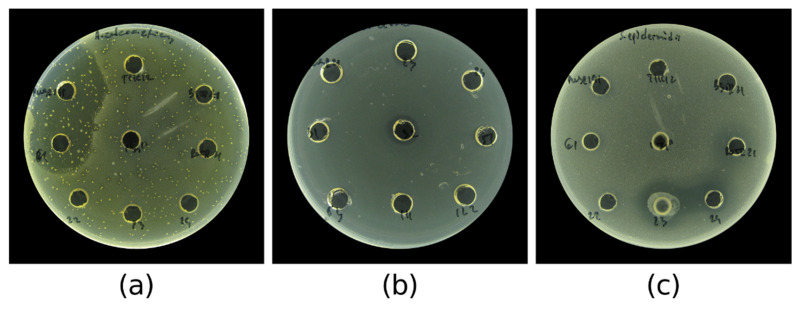
Agar well diffusion assay showing the antibacterial activity of CFSs obtained from the strains against: (**a**) *A. calcoaceticus* ATCC 23055, (**b**) *E. faecalis* ATCC 29212, and (**c**) *S. epidermidis* ATCC 12228.

A crucial step in antibiotics discovery is their mass production in a liquid medium. One of the pitfalls in this process is the inability of the active strains to produce AMC when grown in a liquid culture [7,40]. To verify that the strains are able to produce AMC, which are secreted in the liquid medium, we obtained cell-free supernatants (CFSs) from the 22 confirmed bioactive strains and again evaluated their antibacterial activity by the agar well diffusion assay against the same panel of six test pathogens. This reduced the number of active isolates to 16 (30% from the initial 54), which represented a 0.2% success rate from the initial library of over 7400 colonies. In this assay, the CFSs from the strains were not able to inhibit the growth of *E. cloacae*, *P. putida*, and *E. coli*. However, the inhibitory zones against the rest of the test pathogens varied from 11 mm to 31.5 mm, as could be seen in Table 3. Again, a moderate to strong inhibition was observed against *A. calcoaceticus* (14–31.5 mm). The CFSs showed low antibacterial activity against *E. faecalis* (8–11.5 mm) and *S. epidermidis* (9–16 mm) as could be seen in Figure 6. *Bacillus haynesii* PWSR23, *B. haynesii* PWSR32, *Bacillus paralicheniformis* PWSR191, *B. haynesii* BSTR61, and *V. salarius* POTR191 were recognized as the most potent AMC producers based on the diameter of the inhibitory zones and the number of inhibited test pathogens. Their CFSs were subjected to ethyl acetate extraction, and the antibacterial activity of the extracts was quantitatively assessed by MIC (minimal inhibitory concentration) and MBC (minimal bactericidal concentration) determination.

Some of the strains showed very weak and non-reproducible activity, which was designated as W in the table.

### 2.8. Antibacterial Activity of Ethyl Acetate Extract from V. salarius POTR191

**Figure 7:**
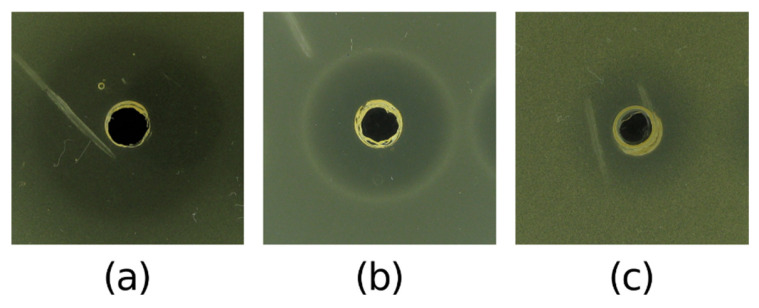
Antibacterial activity of ethyl acetate extract of *V. salarius* POTR191 showing inhibitory zones against (**a**) *A. calcoaceticus* ATCC 23055, (**b**) *E. faecalis* ATCC 29212, and (**c**) *S. epidermidis* ATCC 12228.

From the five selected most potent AMC-producing halophilic strains, only the ethyl acetate extract of *V. salarius* POTR191 demonstrated antibacterial activity in the preliminary agar well diffusion assay. The extract was able to inhibit the growth of three of the test pathogens (Table 4). Strong inhibitory activity was observed against *A. calcoaceticus,* where the inhibitory zone was 27.5 ± 0.5 mm (Figure 7). The extract from *V. salarius* POTR191 showed moderate activity against *E. faecalis* (20.5 ± 0.5 mm) and *S. epidermidis* (14.5 ± 0.5 mm). All of the values were higher than those observed for the CFS of the same strain.

The MIC of the extract was determined via the broth microdilution method [41] as the lowest concentration that visually inhibited the growth of the respective pathogen. MIC values were found to be 128 μg/mL for *A. calcoaceticus* and *E. faecalis*, and 512 μg/mL for *S. epidermidis* (Table 4). After determination of MIC, the MBC was identified by spot plating of 10 μL of MIC and the two adjacent more concentrated dilutions. A MBC of >512 μg/mL was found for all three of the test pathogens.

## 3. Discussion

During the last 40 years, approximately 65% of all discovered antibiotics have been derived from natural products [42]. A statistical review of the scientific literature in the period 1985–2012 found 521 AMC discovered from halophilic marine bacteria [43]. Between 2010 and 2015, they were identified to be over 50 [44], although, until 2011, only 23 AMC were in the preclinical trial stage [45]. Even to this day, the early-stage natural products screening efforts are still somehow financially neglected [46]. Despite that, a main goal of the scientific community should be to assemble a vast collection of already well-characterized bioactive AMC that, when needed, will serve as a foundation for future clinical trials and the final development of commercial antibiotic products. Therefore, the development of novel large-scale screening methods providing rapid and cheap AMC hit discovery would facilitate this research effort.

To address this issue, we combined two old techniques, namely the replica plate assay and the agar overlay assay, in order to establish a high-throughput method for large-scale screening of the antibacterial activity of a library of bacterial isolates. By the use of the newly developed 3D-printed Petri plate replicator, we successfully isolated and screened in parallel over 7400 halophilic bacterial colonies without the need to initially obtain pure cultures. This target-based screening led to a hit rate of 0.7% discovered potential AMC producers. This method, combined with the classic liquid fermentation and agar well diffusion assay, allowed us to confirm 22 potent AMC producers, which were able to inhibit the growth of at least one safe relative of the ESKAPE group pathogens. The CFSs from these strains had a strong inhibitory activity against *A. calcoaceticus* (14–31 mm) and moderate activity against *E. faecalis* (7.5–11.5 mm) and *S. epidermidis* (6–16 mm). Finally, the ethyl acetate extract of *V. salarius* POTR191 demonstrated moderate inhibitory activity against *A. calcoaceticus, E. faecalis*, and *S. epidermidis*, as determined by the MIC values of 128 μg/mL, 128 μg/mL, and 512 μg/mL, respectively.

Three-dimensional printing technologies are just starting to make their way into the microbiology lab, with only a few examples [47]. Nowadays, 3D printers are easily accessible either commercially or for in-house use in the lab [48]. Thus, our newly designed Petri plate replicator could be readily made by any interested researcher without the need to custom purchase special plates, as in other studies [16,18]. With the optimal design of the replicator, up to 74 individual colonies can be inoculated simultaneously on a single plate without prior pure culture isolation, which otherwise significantly slows down the initial stages of AMC discovery. This is due to the fact that each pin of the replicator is designed to hold and deposit 1 μL drops from a sample suspension with a known cell concentration of approximately one cell per microliter, made after a bacterial cell count of the sample and proper dilution of the suspension. After incubation for three days at 35 °C, the colonies derived from the individually deposited cells grew in a grid-like manner (Figure 3a). In this way, the number of screened colonies in one assay increased highly.

Moreover, the closed contact between different colonies co-cultured in one plate has been found to create stimulatory interspecies interactions, which may induce the production of secondary metabolites with antibacterial activity [20]. For the same reason, the bioactive strains were co-cultured with nine strains in one plate during the secondary screening, as proposed by Mohamed et al. (2021) [40]. By the replica plate assay, the strains can be screened for their antagonistic activity against several pathogens at once. Furthermore, the strains could be replicated on several culture media with different content (e.g., low nutrient medium) to study which medium is optimal for the production of AMC. The close contact between the producer and indicator strain could also induce the synthesis of AMC, as discussed by Mohamed et al. (2021) [40]. Nevertheless, in our method, we spatially divided the halophilic producer strains and the indicator pathogen by using two agar layers with different culture media. This was necessary due to the different nutritional and environmental requirements of the two groups of strains. Halophilic bacteria need a specific salt content, which, on the other hand, is harmful for most of the test pathogens used, as mentioned by Sánchez-Hidalgo et al. (2012) [18] (except *S. epidermidis,* which can tolerate up to 10% NaCl [49]). Therefore, in our study, the easier way of co-cultivation on one medium was not applicable, as in other studies [25,40]. However, this enables the screening of a wide range of producer strains falling into different groups of extremophilic or fastidious bacterial species. Our method could be readily applied to thermophilic, psychrophilic, or oligotrophic bacterial strains, which are now extensively researched for the production of novel AMC [50]. This statement is also evident from the temporal division of the producer and indicator strains during the cultivation processes, allowing various regimes of incubation of the target bacteria (such as extremes in temperature, oxygen availability, or pressure) without damaging the vitality and growth of the indicator pathogen and thus compromising the results from the antagonistic assay.

For the early stages of the antibacterial screening, we used a modified agar overlay assay. During the assay, the use of two separate agar layers with different media further ensures the lack of interference between the components of the saline media used for the incubation of the halophilic bacteria (or another kind of extremophiles) and the test pathogens (Muller-Hinton agar). We also added an additional middle thin layer of 0.7% water agar that served two functions—as a buffer layer between the two nutrient media and as a layer in which the colonies are embedded in place. Smear of the producer colonies is a common issue during agar overlay assay, resulting in the overgrowth of the producer strains in the top layer seeded with the test pathogen, thus compromising the study (see Figure S2) [51]. In the past, chloroform vapors were used to kill the producer bacteria prior to the assay [52]. This is a hazardous procedure, and on the other hand, chloroform can easily dissolve or degrade the plastic Petri plates used today. For the top layer, Klein et al. (2020) [51] used a kanamycin-amended overlay medium seeded with a kanamycin-resistant transformant of *Erwinia amylovora* to eliminate the step of vapor killing the producer strains. Other authors [53] flip the agar layer with a sterile spatula and inoculate the pathogen on the reverse side, but according to our trials with this procedure, smear was again detected. Our modification of the agar overlay assay avoided using hazardous chemicals or the need to produce genetically modified target strains. We overcame the issue of colony smear by incubating the Petri plates with grown colonies in a refrigerator, which hardens the bottom agar layer and ensures the rapid solidification of the poured molten water agar in order not to disturb the colonies while, at the same time, embedding them.

In that manner, a single researcher can inoculate several hundred colonies a day. The whole pipeline of the antibacterial screening can be accomplished in four days, excluding the initial isolation. We propose that the 3D-printed replicator could be used for a replica plate of the same strains on different screening media for quantitative investigation of various enzyme activities. Other applications for screening eDNA metagenomic libraries [54] or recombinant mutants by positive selection [55] could also be envisioned.

Despite the high-throughput efficiency of our rapid and large-scale method, we should note some drawbacks. The main limitation is the need for an initial cell count of the culturable bacterial concentration in each of the studied samples. However, with the help of miniaturized cell count assays such as the single plate-serial dilution assay [35], several samples can be plated and enumerated on a single Petri plate, which significantly decreases the time and consumables needed. But, sometimes, deviations from the calculated cell concentration were observed, which led to the growth of two to three colonies from one pin of the replicator (concentration more than 1 cell/μL). If activity is observed from this “spot” containing more than one colony, this could be overcome by retrieving each of those colonies and isolating them in pure cultures by restreaking. The use of nutrient-rich media for routine isolation of halophilic bacteria during the antibacterial screening might not have been the best option. Cultivation of the producer strains on a nutrient-limited medium was found to be more efficient in the discovery of AMC-producing bacteria [40]. Furthermore, our methodology can overlook slow-growing bacteria and those strains producing AMC in low quantities that cannot be detected by the diffusion assay.

Our methodology cannot guarantee the isolation of novel species, but with the use of different culture media and/or different incubation conditions, this could be improved. Finally, the use of our 3D-printed Petri plate replicator might not overcome the limitations detected in previous studies regarding the isolation of already known compounds. In that context, in order to ensure that novel AMC are indeed found, our methodology should be coupled with a chemical analysis of the extracted compounds produced in the CFSs. However, bearing in mind that our workflow enables the isolation of a vast number of strains, the successes of novel AMC discovery may be significantly increased.

The 0.7% hit rate observed in our study is not surprising. It is estimated that the rate of discovery of antimicrobial compounds can be as low as 0.1% [56]. O’Sullivan et al. (2019) [57] screened over 90,000 colonies isolated from 140 swabs from skin microbiota of human patients and found 25 possible bacteriocin producers at a success rate of 0.03%. This only emphasizes the need for a large number of samples and large collections of strains. Our results are consistent with other studies that used replica plate assay (3% [26] and 2% [25] success rate) or agar overlay assay (1.7% identified AMC producers [58]). Among the found potential AMC producers, we observed low species diversity with most of the strains belonging to the genera *Bacillus* and *Virgibacillus*. To the best of our knowledge, the antibacterial activity of *Virgibacillus marismortui* and *Salinivibrio costicola* has not been reported before. Elyasifar et al. (2019) [59] found that *V. olivae* D8B had an inhibitory zone of 29 mm against *Pseudomonas syringae*. On the other hand, *B. haynesii* and *B. licheniformis* are well-known for their antibacterial activity, further supported by our data [60,61]. However, the ethyl acetate extracts from *B. haynesii* PWSR23, *B. haynesii* PWSR32, *B. paralicheniformis* PWSR191, and *B. haynesii* BSTR61 did not exhibit antibacterial activity in comparison with the CFS. This might be attributed to the bioactive compound extraction method. *B. haynesii* is known to produce predominantly bacteriocin-like peptides [61]. The ethyl acetate extraction we used could not be suitable for the proper precipitation of those peptides, although other authors observed that it was the most appropriate method of extraction [61].

## 4. Materials and Methods

### 4.1. Reagents, Chemicals, and Strains

Muller-Hinton broth, Muller-Hinton agar, nutrient broth, bacteriological agar powder, peptone, and yeast extract were purchased from HiMedia (HiMedia Laboratories GmbH, Odenwald str. 18 A, 64,397 Modautal, Germany). All other reagents were purchased from Sigma-Aldrich (Merck KGaA, Darmstadt, Germany) and Thermo Fisher Scientific Inc. (Waltham, MA, USA), unless otherwise stated. *Acinetobacter calcoaceticus* ATCC 23055, *Enterobacter cloacae* ATCC 13047, *Enterococcus faecalis* ATCC 29212, *Escherichia coli* ATCC 25922, *Pseudomonas putida* ATCC 12633, and *Staphylococcus epidermidis* ATCC 12228 were purchased from the Bulgarian National Collection for Microorganisms and Cell Cultures (NBIMCC, Sofia, Bulgaria).

### 4.2. Sampling, Sample Processing, and Culturable Bacteria Cell Enumeration

**Figure 8:**
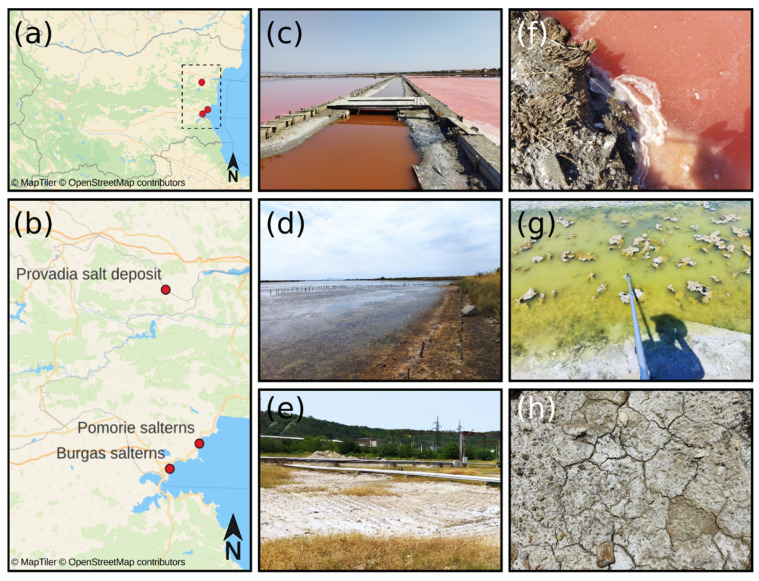
Location of the three hypersaline habitats. (**a**) Map of Bulgaria and (**b**) location of the sampling points: (**c**) Burgas salterns, (**d**) Pomorie salterns, and (**e**) Provadia salt deposit. (**f**–**h**) depict typical samples of brine, saline mud, and saline soil, respectively. The maps were generated with QGIS 3.42.2 ‘Münster’ https://www.qgis.org/ (accessed on 10 June 2025) using the MapTiler layer http://openmaptiles.org/ (accessed on 10 June 2025).

A total of 101 samples were collected from three different hypersaline habitats in Bulgaria—Provadia salt deposit, Burgas salterns, and Pomorie salterns (Figure 8). Sampling took place in the period July–August 2024. The geographical coordinates of the sampling points and the sample type are shown in Table S1. Saline soil samples were collected at a random sampling principle with a sterile soil auger at 0–15 cm depth. The upper 2 cm were discarded to eliminate the presence of allochthonous microflora in the samples. Sampling around the root systems of the plants in the field was avoided. Saline mud samples and brine samples were taken with a water sampling pole equipped with a sterile 100 mL polypropylene container at the end. The temperature, pH, and salinity of the water/brine samples were measured at the field with a portable pH meter (pH CHECK, Carl Roth GmbH Co. KG, Karlsruhe, Germany) and portable handheld refractometer HR-190G (OPTIKA^®^, Ponteranica, Italy), respectively. After collection, all samples were transferred to sterile 50 mL Falcon tubes and transported to the laboratory in a cooler bag (VEVOR^®^ Car Refrigerator 20L, Indespale Technology Limited, Dublin, Ireland) at 4 °C within 6 h after sampling.

In the laboratory, saline soil samples were air-dried in a thermostat at 35 °C overnight. A sterile pestle and mortar were used to grind the samples. After that, the ground samples were sieved through a 2 mm mesh sieve and stored at 4 °C in a refrigerator until used. The saline soil samples’ electrical conductivity and pH were measured after mixing at a 1:5 ratio with distilled water (dH_2_O) according to the following International Organization for Standardization (ISO) protocols—ISO 11265:1994 https://www.iso.org/standard/19243.html (accessed on 6 June 2025) and ISO 10390:2021 https://www.iso.org/standard/75243.html (accessed on 6 June 2025), respectively. Their electrical conductivity was measured with a DiST^®^ 4 conductometer (Hanna Instruments Ltd., Leighton Buzzard, Great Britain). The pH of the soil samples was measured with a pH meter Adwa AD1030 (Szeged, Hungary). Saline mud and brine samples were stored without any processing at 4 °C in a refrigerator until used. The Falcon tubes were loosely capped to prevent the development of anoxic conditions.

The culturable bacteria cell concentration of the collected samples was evaluated by a single plate serial dilution spotting assay [35]. Briefly, a 1 g sample was suspended in 10 mL of the optimal dispersant solution described below. This sample suspension was designated as a stock solution from which dilutions from 10^−1^ to 10^−4^ were made. Then, 10 μL of each dilution was plated in five replicates on agar plates with the respective culture medium. The plates were incubated at 35 °C for 96 h. The grown colonies from each dilution were counted and used for CFU/g or CFU/mL calculation.

### 4.3. Optimization of Cell Extraction Procedure

To achieve maximal cell recovery from the different samples, we initially conducted optimization of the cell extraction from the saline soil and saline mud samples. The physical dispersion method, chemical dispersant, and type of culture medium were varied, changing one factor at a time, while the others remained unchanged. In order to investigate the influence of the physical dispersion method, we employed three different methods: by vortexing for 3 × 1 min, by suspending with an electric drill for 3 × 1 min, and by shaking in an orbital shaker for 1 h and 200 rpm at room temperature (~20–22 °C). Six different chemical dispersants were used to study the optimal cell detachment solution: 0.05% Triton X-100, 0.5% Tween 20, 50 mM sodium pyrophosphate, 0.5% sodium citrate, PBS buffer with 10% NaCl, and 10% NaCl. All solutions used were prepared with dH_2_O as diluent. In sterile 15 mL Falcon tubes, 1 g sample was mixed with 10 mL of one of the different dispersant solutions and vortexed for 3 × 1 min. Finally, different culture media used were: PY medium [39], Rodriguez-Valera medium [37], Halophilic agar M590, HM medium [36], Sehgal and Gibbons media [62], and Caton et al. medium [63]. All culture media were supplemented with 20 g/L agar. In sterile 15 mL Falcon tubes, 1 g sample was mixed with 10 mL sterile 10% NaCl solution and vortexed for 3 × 1 min. For all experiments, the suspension was allowed to stand for 5 min at room temperature for full precipitation of the sediments to the bottom of the tube. Then, 100 μL appropriately diluted cell suspension was used to inoculate agar plates, which were incubated at 35 °C in a Chirana thermostat (Stara Tura, Slovakia) for 72–96 h. After that, the grown colonies were counted, and CFU/g sample values were calculated. The conditions that gave the highest CFU/g sample were chosen as the most optimal for the isolation of halophilic bacteria from the respective sample.

### 4.4. 3D-Printed Petri Plate Replicator

The 3D model of the newly developed 3D-printed Petri plate replicator was designed using the open-source software Blender 4.4.3 (https://docs.blender.org/manual/en/latest/copyright.html, accessed on 2 June 2025). The 3D model was sliced with UltiMaker Cura 5.9.0 (https://ultimaker.com/software/ultimaker-cura/#, accessed on 2 June 2025). The slicing settings were 0.4 mm nozzle diameter, 0.2 mm layer height, 1 mm wall thickness, 1 mm top/bottom thickness, 20% infill, grid infill pattern, 235 °C printing temperature (for PETG) and 210 °C (for PLA), 75 °C bed temperature (for PETG) and 60 °C (for PLA), 120 mm/s printing speed, without supports. The Petri plate replicator was made from polyethylene glycol (PETG) or polylactic acid (PLA) (Spectrum Filaments, Spectrum Group Sp. z o.o., Parkowa 85, Pęcice, Poland). Two varieties of the replicator were made, with plastic and with metal pins. These two different varieties were used at different stages of the primary screening process described below. For the metal pins, M3 × 16 stainless steel hex socket head bolts were used. The models were printed with a 3D printer Creality Ender-3 V3 SE (Shenzhen Creality 3D Technology Co., Ltd., Shenzhen, China). The whole model was printed in around 4 h.

**Figure 9:**
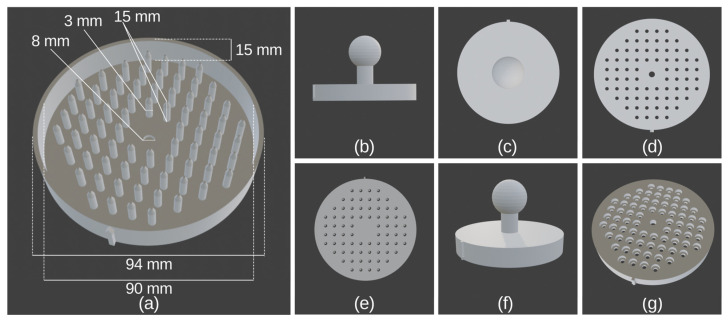
Overall design of the 3D-printed Petri plate replicators with plastic and metal pins. (**a**) General view with dimensions of the plastic pins replicator: (**b**) side view; (**c**) top view; (**e**) bottom view; (**f**) side view, where the marker arrow can be seen. Design of metal pins replicator: (**d**) bottom view with holes for the M3 bolts; (**g**) top view with holes for the heads of the bolts.

The overall design of the 3D-printed Petri plate replicators consisted of a main corpus with 74 plastic or metal pins and a handle (Figure 9). The outer diameter of the corpus was 94 mm, and the inner diameter was 90 mm (Figure 9a). The total corpus height was 15 mm (plastic pins replicator) and 10 mm (metal pins replicator). The basal diameter of the plastic/metal pins was 3 mm while their height was 15 mm and 11 mm, respectively (Figure 9a). For the metal pins, M3 × 16 stainless steel hex socket head bolts were used. The central plate on which the plastic or metal pins were connected was 3 mm and 5 mm, respectively. The holes in the central plate for the metal pins were 3 mm in diameter (Figure 9d). The central four pins were discarded, and, in their place, there was one central hole with a diameter of 8 mm, through which one DIN 933 M8 × 16 bolt was inserted for the mounting of the handle (Figure 9a). The metal pins were installed in specially made holes in the main corpus by a hex screwdriver (Figure 9g). On the corpus, there was a marker arrow, which helps for the proper positioning of the replicator corresponding to the black line on the bottom of the replicated Petri plate (Figure 9f). Photos of the two models of the Petri plate replicator can be seen in Figure S3.

The original 3D models of the different variants of the replicator and the handle are supplied as 3D model files for use with computer-aided design (CAD) software (in “.blend” file format) and as exported stereolithography files (in “.stl” file format) in the Supplementary Materials section. The Petri plate replicator can be viewed and remodeled from the “.blend” files. Anyone interested could directly use the “.stl” files to print the Petri plate replicator.

### 4.5. Method Validation

In order to validate the effectiveness of the newly developed method for screening large libraries of natural isolates for their antibacterial activity, we investigated some parameters of the 3D-printed replicator. Three different replicator designs were made with final pin diameters of 1 mm, 2 mm, and 3 mm. First, the success rate of drop deposition and respective cell inoculation on the agar surface was investigated for the three pin diameters. The volume caught by the pins with different diameters was measured using a 0.1–10 μL automatic pipette. The volume of 20 independent pins was measured. The number of colonies deposited from each pin with a different diameter was also investigated. The colonies inoculated from 20 independent pins were counted and presented as the mean value ± SD. The method of sterilization of the 3D-printed replicator with plastic pins and metal pins was also investigated. Between each replica plate procedure, the plastic pins replicator was immersed in 70% ethanol for 5 s, 10 s, 20 s, 30 s, or 60 s. The metal pins replicator was sterilized by three different methods: immersion in 70% ethanol for 30 s, then immersion in 95% ethanol and one-time flame sterilization; one-time immersion in 70% ethanol and immediate flame sterilization; and three-time immersion in 70% ethanol and immediate flame sterilization. After each sterilization procedure, the sterilized 3D-printed replicator without inoculum was touched to the surface of HM agar plates. After incubation for 72 h at 35 °C, the plates were examined for the presence of growth, which would be an indicator of cross-contamination of the pins. Finally, we studied how many consecutive Petri plates can be replica plated without loss of inoculum and evident contamination.

### 4.6. Simultaneous Isolation and Primary Antibacterial Screening of Halophilic Bacteria

**Figure 10:**
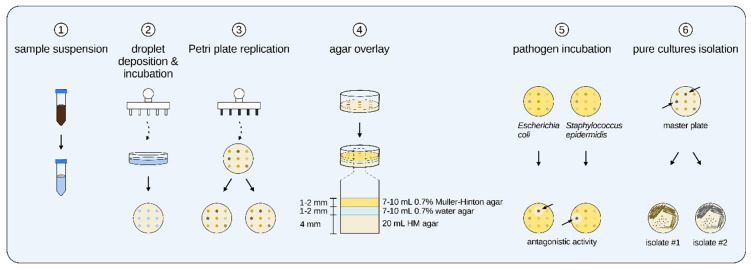
General steps of the primary screening procedure conducted with the newly developed 3D-printed Petri plate replicator. ① The sample was suspended in 10 mL of proper chemical dispersant; ② The plastic pins replicator was immersed in this suspension and droplets were deposited on the HM of Rodriguez-Valera agar surface; ③ After 72 h, the grown colonies were replica plated by the metal pins replicator on two different Petri plates; ④ After another 72 h incubation, the plates were overlayed with semi-solid Muller-Hinton 0.7% agar preinoculated with test pathogen; ⑤ The overlayed Petri plates were incubated for 16–18 h and then inspected for inhibitory zones; ⑥ The strains that showed antagonism were retrieved from the master plate and isolated in pure culture.

The whole workflow of the antibacterial screening aided by the newly developed 3D-printed Petri plate replicator can be seen in Figure 10. Initially, 1 g of sample was mixed with 10 mL of appropriate dispersant solution and vortexed for 3 × 1 min. This suspension was diluted (with a final volume of 10 mL) in order to achieve a cell concentration of ~10^3^ CFU/mL, i.e., approximately one cell per microliter. The diluted cell suspension was poured into a sterile Petri plate. Briefly, the plastic pins replicator was immersed in 70% ethanol for 30 s and then immersed in sterile dH_2_O and washed thoroughly for 3–4 s. The remaining water on the replicator’s pins was dried with sterile filter paper. Then, the droplet replicator was quickly immersed in the Petri plate with the diluted sample and immediately retrieved. After that, the replicator was touched to the surface of HM or Rodriguez-Valera agar plates, which led to the deposition of 74 droplets on the surface of the agar. A black line was drawn on the base of the Petri plate, which corresponded to the position of the marker arrow from the replicator’s corpus. This allowed the proper lining of the replicator to the Petri plate. The Petri plate inoculated in that manner was designated as the master plate and incubated for 72–96 h at 35 °C.

After this initial isolation, the grown colonies on the master plate were replica plated on two Petri plates with the use of the metal pins replicator. The replicator was first immersed in 70% ethanol for 30 s, then immersed in 95% ethanol and one-time flame sterilized. The replicator’s metal pins were first cooled down for 45 s and then touched to the surface of each colony and subsequently touched to the surface of two different agar plates with 20 mL agar media and 4 mm depth. Again, a black line was drawn on the bottom of each replicated plate for the proper positioning of the replicator. The replicated Petri plates were incubated for 72 h at 35 °C. After that, the plates were overlayed by a modified agar overlay assay [64]. Firstly, the replicated plates were incubated for 1 h at 4 °C in a refrigerator. Then, the plates were overlayed with 7–10 mL of 0.7% water agar that was tempered to 42 °C. The plates were again incubated at 4 °C in a refrigerator for 30 min. After that, the plates were overlayed with 7–10 mL semi-solid 0.7% Muller-Hinton agar previously inoculated with either *E. coli* ATCC 25922 or *S. epidermidis* ATCC 12228 to a final cell concentration of 10^6^ CFU/mL. The plates were incubated for 4 h at room temperature (~20–22 °C) for the proper diffusion of the AMC and then incubated for 16–18 h at 37 °C. After the incubation, the plates were screened for inhibitory zones around the colonies with active strains. Those colonies that demonstrated antagonistic activity against one or both of the test pathogens were retrieved from the master plate and isolated in pure culture.

### 4.7. Culture Media and AMC Production by the Active Strains

The isolates that showed antibacterial activity at the primary screening were isolated in pure cultures on HM agar plates. The HM media consisted of: (g/L); NaCl—100; MgSO_4_ × 7H_2_O—10; CaCl_2_ × 2H_2_O—0.36; KCl—2; NaHCO_3_—0.06; NaBr—0.23; FeCl_3_ × 6H_2_O—0.001; peptone—5; yeast extract—10; glucose—1; pH of the medium was adjusted to 7.2 with 1 M NaOH.

For the subsequent antibacterial activity assays, liquid cultures from the strains were obtained. A loopful of each strain was suspended in 500 μL 10% NaCl. This suspension was used for inoculation of 5 mL HM medium in test tubes, which were incubated for 24 h at 35 °C and 180 rpm in a BioSan ES 20/60 orbital shaker (Riga, Latvia). After that, this liquid culture was used as an inoculum for the mass production of AMC. Briefly, 10% inoculum with OD_600_ of 1 was used to inoculate 50 mL HM medium in 100 mL flasks to a final OD_600_ of 0.05. The flasks were cultivated for 72 h at 35 °C and 150 rpm in an orbital shaker. For some of the antibacterial activity assays, cell-free supernatants (CFS) were used. A total of 8 mL liquid cultures were centrifuged at 10,000 rpm for 15 min at 4 °C. The obtained CFS were filter sterilized through a 0.2 μm syringe filter and used on the same day as preparation.

### 4.8. Test Pathogens Cultivation and Inoculum Standardization

The safe relatives of the ESKAPE group pathogens *Acinetobacter calcoaceticus* ATCC 23055, *Enterobacter cloacae* ATCC 13047, *Enterococcus faecalis* ATCC 29212, *Escherichia coli* ATCC 25922, *Pseudomonas putida* ATCC 12633, and *Staphylococcus epidermidis* ATCC 12228 were used as test pathogens. They were grown overnight on Nutrient agar plates at 37 °C. Firstly, a 0.5 McFarland standard suspension was made by suspending several colonies in sterile physiological solution to an OD_600_ of ~0.11. This suspension was diluted 10^−2^ to a final cell concentration of 10^6^ CFU/mL by mixing 1 mL pathogen suspension with 100 mL molten and tempered to 42 °C Muller-Hinton agar.

### 4.9. Secondary Screening of the Antibacterial Activity of the Isolates

Those isolates that demonstrated antibacterial activity at the primary screening were subjected to a secondary screening against *Acinetobacter calcoaceticus* ATCC 23055, *Enterobacter cloacae* ATCC 13047, *Enterococcus faecalis* ATCC 29212, *Escherichia coli* ATCC 25922, *Pseudomonas putida* ATCC 12633, and *Staphylococcus epidermidis* ATCC 12228. Briefly, 20 μL of exponential phase liquid culture from the active strains was spot-inoculated on Petri plates with HM medium. The plates were cultivated for 72 h at 35 °C. After incubation, the plates were overlaid by the same protocol described above and incubated for 16–18 h. The plates were inspected for inhibition zones around the spots with the isolates.

### 4.10. Genomic DNA Extraction, PCR Amplification, and 16S rRNA Gene Sequencing

Total genomic DNA was extracted with the GenElute™ Bacterial Genomic DNA Kit (Merck KGaA, Darmstadt, Germany) following the manufacturer’s instructions. From the isolated genomic DNA, the 16S rRNA gene was amplified by PCR using universal bacterial primers 8F (5′-AGAGTTTGATCCTGG CTCAG-3′) and 1513R (5′-TACGTTACCTTGTTACGA CTT-3′) (SciTechS). The PCR was conducted in BioRAD T100^®^ Thermal Cycler (Bio-Rad Laboratories, Inc., Hercules, CA, USA) with AccuPower^®^ PCR PreMix and Master Mix (Bioneer, Yuseong-gu, Daejeon 34013, Republic of Korea) following the previously mentioned amplification protocol [65]. To verify the presence and integrity of the extracted genomic DNA and amplified 16S rRNA gene PCR products, a 1% agarose gel electrophoresis was conducted with an electrophoretical system Consort E122 (Consort bvba, Turnhout, Belgium). Molecular markers Gene RulerTM 1 kb DNA Ladder (Thermo Fisher Scientific Inc.) were used. The amplified 16S rRNA gene PCR products were commercially sequenced with 27F (5′AGAGTTTGATCMTGGCTCAG-3′) and 1492R (5′ACCTTGTTACGACTT-3′) primers by Macrogen Inc., Seoul, Korea (https://dna.macrogen.com/, accessed on 2 June 2025).

### 4.11. Identification and Phylogenetic Analysis

The AMC producers were taxonomically identified by a BLAST 2.9.0 search [66] based on their 16S rRNA sequences. They were compared to the sequences deposited in the GenBank database in order to find their closest phylogenetic representatives. The isolates were taxonomically assigned to the species with 98% or more sequence similarity. After that, all sequences were retrieved and aligned with the ClustalW algorithm. MEGA11 was used for the reconstruction of the phylogenetic tree by the UPGMA method [67]. The evolutionary distances were computed using the Tamura-Nei method [68] and were in the units of the number of base substitutions per site. The rate variation among sites was modeled with a gamma distribution (shape parameter = 5). The percentage of replicate trees in which the associated taxa clustered together in the bootstrap test (1000 replicates) is shown above the branches [69]. *Thermotoga maritima* SL7 was used to root the tree. All of the nucleotide sequences of the AMC producers were deposited in the GenBank database under the following accession numbers: PV765680.1, PV765681.1, PV765683.1, PV765684.1, PV765686.1, PV765687.1, PV765688.1, PV765692.1, PV765703.1, PV765705.1, PV765707.1, PV765789.1, PV765796.1, PV765822.1, PV765848.1, PV765849.1, PV765883.1, PV765884.1, PV802756.1, PV802757.1, PV802758.1, PV802787.1

### 4.12. Quantitative Assessment of the Antibacterial Activity of the Isolates

The evaluate the presence of AMC in the CFS from the strains exhibiting activity at the secondary screening, we further conducted an agar well diffusion assay [70]. *Acinetobacter calcoaceticus* ATCC 23055, *Enterobacter cloacae* ATCC 13047, *Enterococcus faecalis* ATCC 29212, *Escherichia coli* ATCC 25922, *Pseudomonas putida* ATCC 12633, and *Staphylococcus epidermidis* ATCC 12228 were used as test pathogens. Using a sterile cork borer, 6 mm wells were made in preinoculated Muller-Hinton agar plates in which 100 μL CFS from each strain was pipetted. The plates were incubated for 4 h at room temperature (~20–22 °C) and then incubated at 37 °C for 16–18 h. After that, the plates were observed for inhibition zones without pathogen growth. The antibacterial activity of the CFSs was interpreted by the diameter of the inhibitory zones as follows: <15 mm—low; 15–25 mm—moderate; >25 mm—strong.

### 4.13. Ethyl Acetate Extraction of AMC from V. salarius POTR191

A loopful of strain *V. salarius* POTR191, chosen as the most potent AMC producer, was suspended in 1 mL of 10% NaCl. This suspension was inoculated in 10 mL HM medium and incubated overnight at 35 °C and 180 rpm in an orbital shaker. After that, 10% inoculum was used to inoculate 100 mL HM medium in a 300 mL flask and incubated at 35 °C for 72 h and 150 rpm. The liquid culture was centrifuged at 3200 g for 20 min. The resulting CFS was subjected to liquid-liquid extraction by mixing with an equal volume of ethyl acetate and incubated for 2 h at 35 °C and 180 rpm in an orbital shaker. Then, the organic and aqueous layers were separated with a separating funnel. The aqueous layer was discarded. The organic fraction layer was stored for 24 h at 4 °C in a refrigerator and then evaporated under reduced pressure and temperature of 40 °C with a Rotavapor^®^ R3 rotary vacuum evaporator (BÜCHI Labortechnik AG, Meierseggstrasse 40, Flawil, Switzerland). The resulting solid extract was stored at 4 °C for 24 h before use.

### 4.14. Antibacterial Activity of the Extracted AMC from V. salarius POTR191

The ethyl acetate extract was dissolved in 1% DMSO at a concentration of 5 mg/mL and then filter sterilized through a 0.2 μm syringe filter. The antibacterial activity of the extract was evaluated by the agar well diffusion assay [70] against *Acinetobacter calcoaceticus* ATCC 23055, *Enterococcus faecalis* ATCC 29212, and *Staphylococcus epidermidis* ATCC 12228. The Petri plates were incubated for 2 h at room temperature (~20–22 °C) for proper diffusion of the AMC and then incubated at 37 °C for 16–18 h, and the resulting inhibitory zones were measured in mm.

### 4.15. Minimal Inhibitory and Minimal Bactericidal Concentration of the Extract

The minimal inhibitory concentration (MIC) was determined by broth microdilution assay [41]. From the initial 5 mg/mL stock solution of AMC extract 2048 μg/mL solution was made with dH_2_O. After that, a 2-fold dilution series was made in Muller-Hinton broth till 2 μg/mL. The entire dilution series was made in 2× concentration of the final AMC concentration used in the assay. In 96 well plates, 100 μL of each 2× solution was pipetted in each well, followed by 100 μL Muller-Hinton broth previously inoculated with 10^6^ CFU/mL of the respective pathogen. In that manner, the final concentration of the pathogen was 5 × 10^5^ CFU/mL. As positive growth control, only 200 μL preinoculated Muller-Hinton broth was used. As negative sterility control, only 200 μL uninoculated Muller-Hinton broth was used. The 96-well plates were incubated at 37 °C for 18 h under static conditions, and, after that, were visually observed for lack of growth. The MIC was defined as the lowest concentration to visually inhibit the growth of the respective pathogen.

To determine the minimal bactericidal concentration (MBC), 10 μL from the well with that dilution representing MIC and the following two more concentrated dilutions were plated on Muller-Hinton agar plates [71]. Five replicates were made from each dilution. The plates were then incubated at 37 °C for 18 h. The MBC was defined as the concentration that reduced the growth of the respective pathogen at least by 99.9%.

### 4.16. Statistical Analysis

Each experiment was conducted at least in triplicate unless otherwise stated. The results were presented as their mean value ± standard deviation. The statistical analysis was performed in LibreOffice Calc (The Document Foundation, libreoffice.org). The data visualization was done by SciDAVis (https://scidavis.sourceforge.net/, accessed on 2 June 2025).

## 5. Conclusions

The urgent need for large-scale screening platforms for antibiotic discovery is evident from global projects such as the Tiny Earth project [72] and the Small World Initiative [73]. To address this issue, we developed a high-throughput screening method employing a newly designed 3D-printed replica plate device. The use of this device, coupled with a modified agar overlay assay, allowed the parallel isolation and screening of more than 7400 halophilic bacterial colonies. As a result, a total of 54 potential AMC producers were identified at a success rate of 0.7%. From them, the cell-free supernatants of 22 strains demonstrated antibacterial activity against one or more safe relatives of the ESKAPE group pathogens. The ethyl acetate extract from the prioritized strain *V. salarius* POTR191 manifested moderate MICs of 128 μg/mL, 128 μg/mL, and 512 μg/mL against *Enterococcus faecalis*, *Acinetobacter baumanii*, and *Staphylococcus epidermidis*, respectively. The MBC observed against all three pathogens was higher, with a value of 512 μg/mL. Future experiments are still needed with *V. salarius* POTR191 to find the optimal conditions for AMC production. Moreover, the extracted antibacterial substance(s) should be purified and subjected to dereplication by proper chemical analysis in order to verify their novelty.

## Figures and Tables

**Table 1:** Some physicochemical parameters and culturable bacteria cell count of the samples collected from the three hypersaline environments. The CFU/g sample was calculated for 1 g of saline soil or saline mud. The CFU/mL sample was calculated for 1 mL of brine.

Sampling Point	Temperature (°C)	Salinity (% or dS/m)	pH	CFU/g Sample	CFU/mL Sample
Provadia salt deposit					
South zone	34.0 ± 2.0	7.26 ± 0.24 *	8.2 ± 0.0	3.08 ± 4.56 × 10^4^	—
East zone	34.0 ± 2.0	2.93 ± 0.04 *	8.3 ± 0.1	1.97 ± 2.96 × 10^4^	—
Crystallizer pond	34.0 ± 2.0	20.30 ± 0.61 *	8.0 ± 0.0	0.37 ± 0.08 × 10^4^	—
Burgas salterns					
Feeding pond #1	35.9 ± 0.8	9.0 ± 0.0	8.2 ± 0.0	4.37 ± 3.40 × 10^5^	4.55 ± 5.73 × 10^5^
Feeding pond #2	36.9 ± 1.1	9.3 ± 0.5	8.2 ± 0.0	8.08 ± 5.81 × 10^5^	1.41 ± 0.55 × 10^5^
Feeding pond #3	38.0 ± 0.9	9.8 ± 0.4	8.2 ± 0.0	1.66 ± 2.37 × 10^5^	80.00 ± 17.32 × 10^5^
Feeding pond #4	36.7 ± 0.2	7.3 ± 0.8	8.0 ± 0.1	1.95 ± 3.62 × 10^5^	—
Crystallizer pond #1	35.4 ± 1.2	20.9 ± 8.7	7.5 ± 0.5	4.90 ± 5.45 × 10^5^	1.26 ± 2.34 × 10^5^
Crystallizer pond #2	38.0 ± 0.0	29.0 ± 0.0	7.0 ± 0.0	7.50 ± 3.25 × 10^5^	0.01 ± 0.01 × 10^5^
Pomorie salterns					
Pomorie lake	25.8 ± 0.7	5.9 ± 1.3	9.0 ± 0.2	5.47 ± 3.97 × 10^4^	9.10 ± 5.83 × 10^4^
Feeding pond #1	26.7 ± 0.6	13.8 ± 6.1	7.9 ± 0.5	8.49 ± 1.04 × 10^4^	11.20 ± 1.13 × 10^4^
Feeding pond #2	28.0 ± 0.0	10.0 ± 0.0	8.2 ± 0.0	3.15 ± 2.49 × 10^4^	—
Crystallizer pond	27.7 ± 0.0	27.0 ± 0.0	7.8 ± 0.0	3.36 ± 2.30 × 10^4^	—

* Note that only the salinity of the soil samples from the Provadia salt deposit was expressed in dS/m.

**Table 2:** Testing the most suitable pin diameter of the replicator for proper drop deposition and successful colony growth.

Pin Diameter (mm)	Volume Deposited (μL)	Number of Grown Colonies	Success Rate (%)
1	0.5 ± 0.1	1.04 ± 0.03	88 ± 12
2	1.0 ± 0.1	1.17 ± 0.50	92 ± 9
3	2.2 ± 0.2	2.38 ± 0.31	100 ± 0

**Table 3:** Antibacterial activity of the CFSs obtained from the strains assessed by the agar well diffusion assay. *E. cloacae*, *P. putida*, and *E. coli* were not shown because none of the strains demonstrated activity against them. HM medium was used as a negative control, and 25 μg/mL chloramphenicol was used as a positive control.

	Inhibitory Zone (mm)		
Strain	*A. calcoaceticus* ATCC 23055	*E. faecalis* ATCC 29212	*S. epidermidis* ATCC 12228
*Bacillus haynesii* PWSR21	17 ± 1	8 ± 0	−
*Bacillus haynesii* PWSR23	29.5 ± 0.5	9.5 ± 0.5	−
*Bacillus licheniformis* PWSR24	−	−	−
*Bacillus haynesii* PWSR32	30.5 ± 1.5	11.5 ± 0.5	W
*Bacillus haynesii* PWSR51	14 ± 1	8 ± 0	−
*Bacillus haynesii* PWSR53	17.5 ± 0.5	8 ± 0	−
*Bacillus licheniformis* PWSR111	−	−	−
*Bacillus paralicheniformis* PWSR191	31.5 ± 1.5	9.5 ± 0.5	
*Bacillus altitudinis* T1K12	−	−	−
*Salinivibrio kushneri* BSTR21	−	−	−
*Bacillus haynesii* BSTR61	22.5 ± 2.5	7.5 ± 0.5	−
*Salinivibrio costicola* BSTR171	−	−	−
*Virgibacillus olivae* POTR21	−	−	11 ± 1
*Virgibacillus olivae* POTR22	−	−	−
*Virgibacillus olivae* POTR23	−	−	16 ± 1
*Virgibacillus olivae* POTR24	−	−	11.5 ± 0.5
*Virgibacillus olivae* POLR81	−	−	10.5 ± 0.5
*Virgibacillus olivae* POLR82	−	−	9.5 ± 0.5
*Virgibacillus olivae* POTR122	−	−	W
*Virgibacillus olivae* POTR181	−	−	9 ± 1
*Virgibacillus marismortui* POTR183	−	−	−
*Virgibacillus salarius* POTR191	20.5 ± 0.5	10 ± 0.5	6 ± 0.5
HM medium	0	0	0
chloramphenicol	41.5 ± 1.5	16.5 ± 3.5	26.5 ± 1.5

**Table 4:** MIC and MBC values of the ethyl acetate extract from *V. salarius* POTR191. The antibacterial activity of the extract was also tested by agar well diffusion assay.

Test Pathogen	Inhibitory Zone (mm)	MIC (μg/mL)	MBC (μg/mL)
*A. calcoaceticus* ATCC 23055	27.5 ± 0.5	128	>512
*E. faecalis* ATCC 29212	20.5 ± 0.5	128	>512
*S. epidermidis* ATCC 12228	14.5 ± 0.5	512	>512

## Data Availability

Data is contained within the article or Supplementary Materials. The 16S rRNA gene sequences of the strains are available in a publicly accessible repository (GenBank).

## Supplementary Materials

The following supporting information can be downloaded at: https://www.mdpi.com/article/10.3390/md23080295/s1, Figure S1: Reconstructed phylogenetic tree showing the phylogenetic relationship of the AMC producers with their closest relatives based on the obtained 16S rRNA gene sequences. To reconstruct the tree UPGMA method was used. The optimal tree was shown based on 1000 bootstrap replications, and the bootstrap values of ≥70% were shown above the branches. In order to root the tree, *Thermotoga maritima* SL7 was used as an outgroup.; Figure S2: Colony smear as a result of failed agar overlay assay. The middle layer of 0.7% water agar was omitted. (a) colony smear in the top layer of Muller-Hinton agar, where the producer halophilic strains have outgrown the indicator pathogen; (b) colony smear in the top layer where the producer strains and indicator pathogen have grown together.; Figure S3: General structure of the Petri plate replicators with plastic (top row) and metal (bottom row) pins: (a) replicator’s body and handle, (b), (c) plastic pins with 2 mm diameter, (d) closer view of the plastic pins; note that the pins are beveled at the top, (e) replicator’s body and handle; the holes for the M3 bolts can be seen, (f), (g) metal M3 × 16 bolts, (h) close view of the ends of the M3 bolt with 3 mm diameter.; Table S1: Detailed data of some of the physicochemical characteristics and the culturable bacteria count of the samples.; Table S2: Halophilic bacterial strains recognized as AMC producers during the primary screening with the newly developed 3D-printed Petri plate replicator. The strains were taxonomically identified on the basis of the sequence of the 16S rRNA gene and affiliated with their closest relatives deposited in GenBank.
