# Supplementary material for: Novel 3D-Printed Replica Plate Device Ensures High-Throughput Antibacterial Screening of Halophilic Bacteria
Source: Mar Drugs. 2025 Jul 23;23(8):295. doi: 10.3390/md23080295 (PMC12387310; doi:10.3390/md23080295)
Supplement: Supplementary file 1 [file marinedrugs-23-00295-s001.zip › marinedrugs-3767148-Supplementary_Materials/Supplementary_materials.pdf]

Supplementary Materials

Novel 3D-printed replica plate device ensures high-throughput antibacterial screening of halophilic bacteria

Kaloyan Berberov [1,*], Nikolina Atanasova [1], Nikolay Krumov [1], Boryana Yakimova [2,3], Irina Lazarkevich [1], Stephan Engibarov [1], Tsvetozara Damyanova [1], Ivanka Boyadzhieva [1] and Lyudmila Kabaivanova [4]

[1] Department of General Microbiology, The Stephan Angeloff Institute of Microbiology, Bulgarian Academy of Sciences, Sofia 1113, Bulgaria, kberberov@microbio.bas.bg (K.B.); nikolina@microbio.bas.bg (N.A.); krumov.1993@gmail.com (N.K.); irinalazarkevich@abv.bg (I.L.); stefan_engibarov@abv.bg (S.E.); tsvetozaradamianova@gmail.com (T.D.); petrovaim@abv.bg (I.B.)
[2] Laboratory of Chemistry and Biophysics of Proteins and Enzymes, Institute of Organic Chemistry with Centre of Phytochemistry, Bulgarian Academy of Sciences, 1113, Sofia, Bulgaria; boryana.yakimova@orgchm.bas.bg (B.Y.)
[3] Institute of Neurobiology, Bulgarian Academy of Sciences, 1113, Sofia, Bulgaria; boryana.yakimova@orgchm.bas.bg (B.Y.) ,
[4] Department of Biotechnology, The Stephan Angeloff Institute of Microbiology, Bulgarian Academy of Sciences, Sofia 1113, Bulgaria lkabaivanova@microbio.bas.bg (L.K.)
* Correspondence: kberberov@microbio.bas.bg

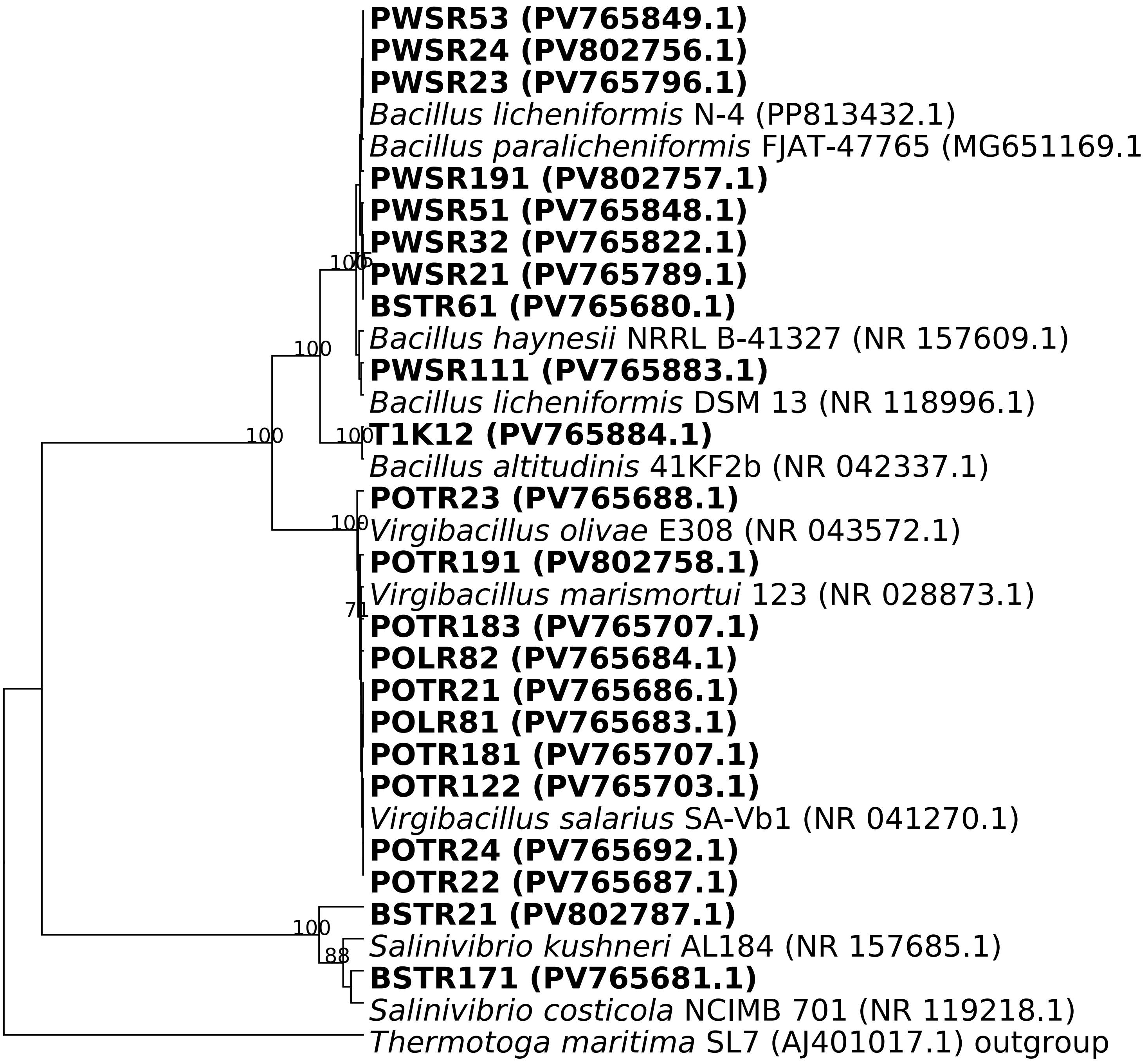

Figure S1. Reconstructed phylogenetic tree showing the phylogenetic relationship of the AMC producers with their closest relatives based on the obtained 16S rRNA gene sequences. To reconstruct the tree UPGMA method was used. The optimal tree was shown based on 1000 bootstrap replications and the bootstrap values of ≥70% were shown above the branches. In order to root the tree *Thermotoga maritima* SL7 was used as an outgroup.

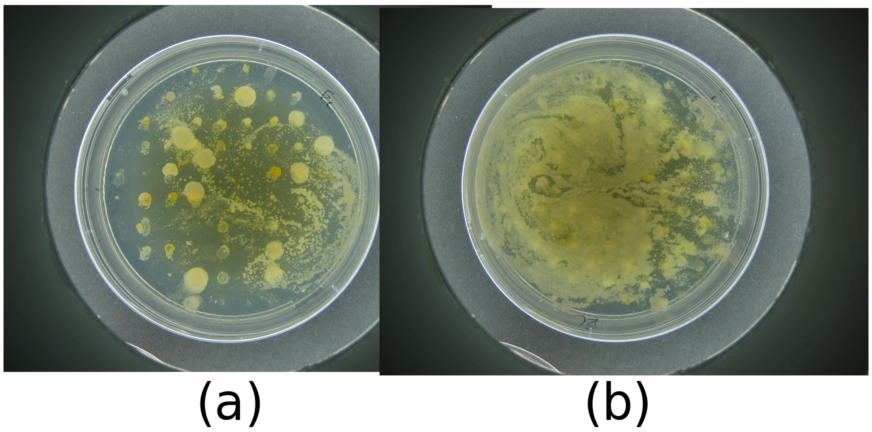

Figure S2. Colony smear as a result of failed agar overlay assay. The middle layer of 0.7% water agar was omitted. **(a)** colony smear in the top layer of Muller-Hinton agar where the producer halophilic strains have outgrown the indicator pathogen; **(b)** colony smear in the top layer where the producer strains and indicator pathogen have grown together.

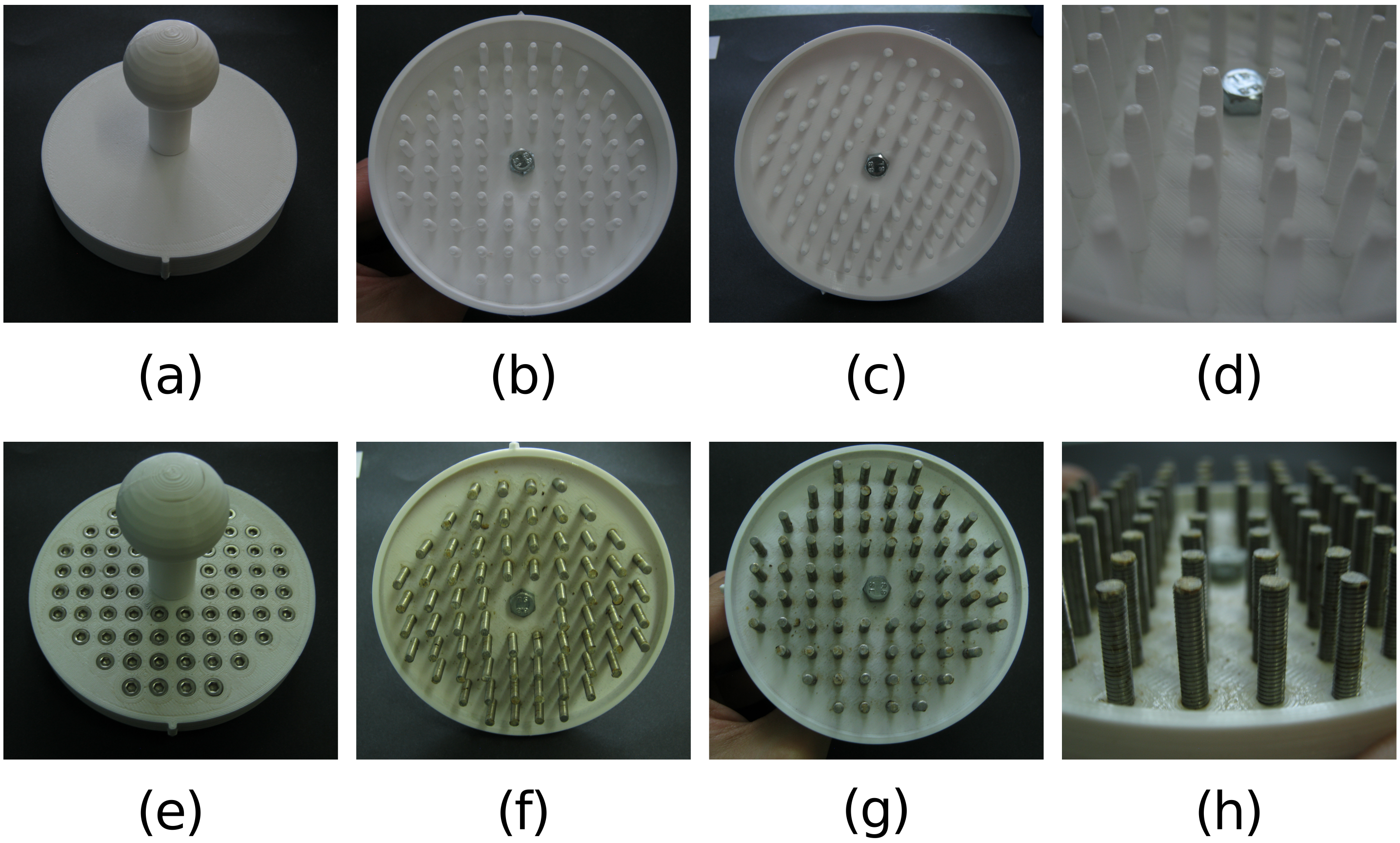

Figure S3. General structure of the Petri plate replicators with plastic (top row) and metal (bottom row) pins: **(a)** replicator's body and handle, **(b)**, **(c)** plastic pins with 2 mm diameter, **(d)** closer view of the plastic pins; note that the pins are beveled at the top, **(e)** replicator's body and handle; the holes for the M3 bolts can be seen, **(f)**, **(g)** metal M3x16 bolts, **(h)** close view of the ends of the M3 bolt with 3 mm diameter.

Table S1. Detailed data of some of the physicochemical characteristics and the culturable bacteria count of the samples.

Locality	Sampling point	Coordinates	Sample ID	Sample type	Temperature	Salinity	pH	CFU/g sample or CFU/mL sample
Provadia salt deposit	South zone	43°07'39.4"N 27°28'22.3"E	PWS1	saline soil	34 ºC	7.26 ± 0.24 dS/m	8.2	1.40 ± 0.39x10^{4}
			PWS2	saline soil	34 ºC	7.26 ± 0.24 dS/m	8.2	0.70 ± 0.16x10^{4}
			PWS3	saline soil	34 ºC	7.26 ± 0.24 dS/m	8.2	0.56 ± 0.69x10^{4}
			PWS4	saline soil	34 ºC	7.26 ± 0.24 dS/m	8.2	1.54 ± 0.77x10^{4}
			PWS5	saline soil	34 ºC	7.26 ± 0.24 dS/m	8.2	20.80 ± 1.64x10^{4}
			PWS6	saline soil	34 ºC	7.26 ± 0.24 dS/m	8.2	11.20 ± 5.26x10^{4}
	Crystaliser pond	43°07'41.0"N 27°28'25.9"E	PWS7	saline mud	34 ºC	20.30 ± .0.61 dS/m	8.0	0.42 ± 0.16x10^{4}
			PWS8	saline mud	34 ºC	20.30 ± .0.61 dS/m	8.0	0.40 ± 0.24x10^{4}
			PWS9	saline mud	34 ºC	20.30 ± .0.61 dS/m	8.0	0.28 ± 0.28x10^{4}
	East zone	43°08'07.5"N 27°28'29.1"E	PWS10	saline soil	34 ºC	2.93 ± 0.04 dS/m	8.2	1.24 ± 0.53x10^{4}
			PWS11	saline soil	34 ºC	2.93 ± 0.04 dS/m	8.2	3.52 ± 0.65x10^{4}
			PWS12	saline soil	34 ºC	2.93 ± 0.04 dS/m	8.2	8.80 ± 2.49x10^{4}
			PWS13	saline soil	34 ºC	2.93 ± 0.04 dS/m	8.2	0.18 ± 0.15x10^{4}
			PWS14	saline soil	34 ºC	2.93 ± 0.04 dS/m	8.2	1.68 ± 0.50x10^{4}
			PWS15	saline soil	34 ºC	2.93 ± 0.04 dS/m	8.2	0.96 ± 0.13x10^{4}
			PWS16	saline soil	34 ºC	2.93 ± 0.04 dS/m	8.2	0.26 ± 0.11x10^{4}
			PWS17	saline soil	34 ºC	2.93 ± 0.04 dS/m	8.2	0.6 ± 0.35x10^{4}
	East zone	43°08'03.6"N 27°28'32.1"E	PWS18	saline soil	34 ºC	2.93 ± 0.04 dS/m	8.45	1.14 ± 0.26x10^{4}
			PWS19	saline soil	34 ºC	2.93 ± 0.04 dS/m	8.45	2.28 ± 1.05x10^{4}
			PWS20	saline soil	34 ºC	2.93 ± 0.04 dS/m	8.45	0.70 ± 0.29x10^{4}
			PWS21	saline soil	34 ºC	2.93 ± 0.04 dS/m	8.45	0.12 ± 0.11x10^{4}
			PWS22	saline soil	34 ºC	2.93 ± 0.04 dS/m	8.45	2.58 ± 0.50x10^{4}
			PWS23	saline soil	34 ºC	2.93 ± 0.04 dS/m	8.45	0.52 ± 0.33x10^{4}
			PWS24	saline soil	34 ºC	2.93 ± 0.04 dS/m	8.45	0.52 ± 0.13x10^{4}
			PWS25	saline soil	34 ºC	2.93 ± 0.04 dS/m	8.45	1.26 ± 0.17x10^{4}
			PWS26	saline soil	34 ºC	2.93 ± 0.04 dS/m	8.45	11.4 ± 3.71x10^{4}
			PWS27	saline soil	34 ºC	2.93 ± 0.04 dS/m	8.45	0.60 ± 0.25x10^{4}
			PWS28	saline soil	34 ºC	2.93 ± 0.04 dS/m	8.45	0.16 ± 0.13x10^{4}
			PWS29	saline soil	34 ºC	2.93 ± 0.04 dS/m	8.45	0.30 ± 0.07x10^{4}
			PWS30	saline soil	34 ºC	2.93 ± 0.04 dS/m	8.45	2.28 ± 0.68x10^{4}
Burgas salterns	Crystaliser pond #1	42.529845º N 27.487366º E	BST1	saline mud	34 ºC	≥29%	7.0	0.04 ± 0.01x10^{5}
			BSL1	lye	34 ºC	≥29%	7.0	0.01 ± 0.01x10^{5}
			BSL2	lye	36.7 ºC	10%	8.0	0.85 ± 0.31x10^{5}
			BST2	saline mud	36.7 ºC	10%	8.0	9.00 ± 1.22x10^{5}
			BSL3	lye	35.8 ºC	9%	8.0	5.40 ± 1.52x10^{5}
			BSL4	lye	35.4 ºC	29%	6.9	0.01 ± 0.01x10^{5}
			BST4	saline mud	36.4 ºC	24%	7.4	10.20 ± 4.44x10^{5}
			BSL5	lye	36.4 ºC	24%	7.4	0.02 ± 0.01x10^{5}
			BST5	saline mud	36.8 ºC	24%	7.5	0.36 ± 0.17x10^{5}
	Feeding pond #1	42.530509º N 27.486317º E	BST6	saline mud	36.2 ºC	9%	8.2	4.40 ± 1.14x10^{5}
			BST7	saline mud	36.2 ºC	9%	8.2	0.96 ± 0.34x10^{5}
			BST8	saline mud	34.9 ºC	9%	8.2	7.75 ± 2.99x10^{5}
			BST9	saline mud	34.9 ºC	9%	8.2	50.00 ± 11.55x10^{5}
			BSL7	lye	34.9 ºC	9%	8.2	0.50 ± 0.19x10^{5}
			BSL6	lye	36.2 ºC	9%	8.2	8.60 ± 1.95x10^{5}
	Feeding pond #2	42.529619º N 27.485899º E	BSL8	lye	36.2 ºC	9%	8.2	5.00 ± 1.87x10^{5}
			BST10	saline mud	36.2 ºC	9%	8.2	12.40 ± 6.39x10^{5}
			BST11	saline mud	36.2 ºC	9%	8.2	0.45 ± 0.13x10^{5}
			BST12	saline mud	36.2 ºC	9%	8.2	12.80 ± 2.17x10^{5}
			BST13	saline mud	36.2 ºC	9%	8.2	88.00 ± 29.5x10^{5}
			BST14	saline mud	36.2 ºC	9%	8.2	6.67 ± 0.58x10^{5}

**Burgas salterns**	Feeding pond #2	42.529619º N 27.485899º E	BSL9	lye	38.3 ºC	10%	8.2	$1.02 \pm 0.38x10^{5}$
			BSL10	lye	38.3 ºC	10%	8.2	**ND**
			BSL11	lye	38.3 ºC	10%	8.2	$1.80 \pm 0.32x10^{5}$
	Feeding pond #3	42.531490º N 27.489777º E	BST15	saline mud	38.3 ºC	10%	8.2	$5.20 \pm 2.59x10^{5}$
			BST16	saline mud	38.3 ºC	10%	8.2	$0.72 \pm 0.15x10^{5}$
			BST17	saline mud	38.3 ºC	10%	8.2	$0.28 \pm 0.04x10^{5}$
			BST18	saline mud	38.3 ºC	10%	8.2	$198.00 \pm 34.21x10^{5}$
			BST19	saline mud	38.3 ºC	10%	8.2	$0.43 \pm 0.06x10^{5}$
			BSL12	lye	36.2 ºC	9%	8.2	$80.00 \pm 17.32x10^{5}$
	Feeding pond #4	42.532082º N 27.484725º E	BST20	saline mud	36.2 ºC	9%	8.2	$0.01 \pm 0.01x10^{5}$
			BST21	saline mud	36.8 ºC	7%	7.9	$0.52 \pm 0.15x10^{5}$
			BST22	saline mud	36.8 ºC	7%	7.9	$0.80 \pm 0.21x10^{5}$
			BST23	saline mud	36.8 ºC	7%	7.9	$0.01 \pm 0.01x10^{5}$
			BST24	saline mud	36.8 ºC	7%	7.9	$0.70 \pm 0.24x10^{5}$
			BST25	saline mud	36.8 ºC	7%	7.9	$8.40 \pm 0.89x10^{5}$
	Crystaliser ponds #2	42.532450º N 27.485005º E	BST26	saline mud	38 ºC	≥29%	7.0	$9.80 \pm 2.49x10^{5}$
			BST27	saline mud	38 ºC	≥29%	7.0	$5.20 \pm 1.92x10^{5}$
			BSL13	lye	38 ºC	≥29%	7.0	$0.01 \pm 0.01x10^{5}$
**Pomorie salterns**	Feeding pond #1	42.616403º N 27.630074º E	POL9	lye	26.8 ºC	18%	7.6	**ND**
			POT10	saline mud	26.8 ºC	18%	7.6	$87.60 \pm 3.21x10^{4}$
			POL10	lye	27.4 ºC	17.5%	7.6	$10.40 \pm 3.44x10^{4}$
			POT1	saline mud	27.4 ºC	17.5%	7.6	$9.22 \pm 0.19x10^{4}$
			POL5	lye	26 ºC	6%	8.5	$12.00 \pm 2.35x10^{4}$
			POT18	saline mud	26 ºC	6%	8.5	$7.75 \pm 2.06x10^{4}$
	Pomorie lake	42.613118º N 27.627714º E	POL8	lye	25.8 ºC	5%	8.8	$16.40 \pm 1.52x10^{4}$
			POT11	saline mud	25.8 ºC	5%	8.8	$8.80 \pm 3.70x10^{4}$
			POT12	saline mud	25.8 ºC	5%	8.8	$10.60 \pm 2.07x10^{4}$
			POL3	lye	25.6 ºC	8%	8.8	$11.20 \pm 3.35x10^{4}$
			POT2	saline mud	25.6 ºC	8%	8.8	$2.72 \pm 1.00x10^{4}$
			POL2	lye	25.7 ºC	5.5%	9.0	$4.20 \pm 3.11x10^{4}$
			POT19	saline mud	25.7 ºC	5.5%	9.0	$3.60 \pm 1.14x10^{4}$
			POL7	lye	25.2 ºC	5%	9.3	$4.60 \pm 1.34x10^{4}$
			POT20	saline mud	25.2 ºC	5%	9.3	$1.64 \pm 0.26x10^{4}$
	Crystaliser pond	42.614965º N 27.628166º E	POL6	lye	27.7 ºC	27%	7.8	**ND**
			POT29	saline mud	27.7 ºC	27%	7.8	**ND**
			POT28	saline mud	27.7 ºC	27%	7.8	$4.98 \pm 0.98x10^{4}$
			POT30	saline mud	27.7 ºC	27%	7.8	$1.73 \pm 0.29x10^{4}$
	Feeding pond #2	42.614327º N 27.626330º E	POT22	saline mud	28 ºC	10%	8.2	$4.62 \pm 1.13x10^{4}$
			POT26	saline mud	28 ºC	10%	8.2	**ND**
			POT4	saline mud	28 ºC	10%	8.2	**ND**
			POT14	saline mud	28 ºC	10%	8.2	**ND**
			POT17	saline mud	28 ºC	10%	8.2	$0.24 \pm 0.13x10^{4}$
			POT27	saline mud	28 ºC	10%	8.2	**ND**
			POT7	saline mud	28 ºC	10%	8.2	$5.96 \pm 0.30x10^{4}$
			POT6	saline mud	28 ºC	10%	8.2	$0.84 \pm 0.26x10^{4}$
			POT15	saline mud	28 ºC	10%	8.2	**ND**
			POT13	saline mud	28 ºC	10%	8.2	**ND**
			POT8	saline mud	28 ºC	10%	8.2	**ND**
			POT16	saline mud	28 ºC	10%	8.2	$4.10 \pm 0.29x10^{4}$
			POT9	saline mud	28 ºC	10%	8.2	**ND**

ND – Not determined

Table S2. Halophilic bacterial strains recognised as AMC producers during the primary screening with the newly developed 3D-printed Petri plate replicator. The strains were taxonomically identified on the basis of the sequence of the 16S rRNA gene and affiliated to their closest relatives deposited in GenBank.

Strain ID	Acc. Num.	Closest relative in GenBank	Length (bp)	Per. Identity	Acc. Len.
PWSR21	PV765789.1	*Bacillus haynesii* NRRL B-41327 (NR_157609.1)	1405	99.43%	1508
PWSR23	PV765796.1	*Bacillus haynesii* NRRL B-41327 (NR_157609.1)	997	99.50%	1508
PWSR24	PV802756.1	*Bacillus licheniformis* N-4 (PP813432.1)	1343	100.00%	1467
PWSR32	PV765822.1	*Bacillus haynesii* NRRL B-41327 (NR_157609.1)	1401	99.43%	1508
PWSR51	PV765848.1	*Bacillus haynesii* NRRL B-41327 (NR_157609.1)	138	99.78%	1508
PWSR53	PV765849.1	*Bacillus haynesii* NRRL B-41327 (NR_157609.1)	1383	99.71%	1508
PWSR111	PV765883.1	*Bacillus licheniformis* DSM 13 (NR_118996.1)	1375	99.85%	1545
PWSR191	PV802757.1	*Bacillus paralicheniformis* FJAT-47765 (MG651169.1)	1375	99.93%	1422
T1K12	PV765884.1	*Bacillus altitudinis* 41KF2b (NR_042337.1)	1349	99.78%	1506
BSTR21	PV802787.1	*Salinivibrio kushneri* AL184 (NR_157685.1)	1408	98.10%	1557
BSTR61	PV765680.1	*Bacillus haynesii* NRRL B-41327 (NR_157609.1)	1402	99.50%	1508
BSTR171	PV765681.1	*Salinivibrio costicola* NCIMB 701 (NR_119218.1)	1049	100.00%	1511
POTR21	PV765686.1	*Virgibacillus olivae* E308 (NR_043572.1)	1394	99.28%	1564
POTR22	PV765687.1	*Virgibacillus olivae* E308 (NR_043572.1)	1411	99.58%	1564
POTR23	PV765688.1	*Virgibacillus olivae* E308 (NR_043572.1)	1397	99.21%	1564
POTR24	PV765692.1	*Virgibacillus olivae* E308 (NR_043572.1)	1403	99.50%	1564
POLR81	PV765683.1	*Virgibacillus olivae* E308 (NR_043572.1)	1416	99.72%	1563
POLR82	PV765684.1	*Virgibacillus olivae* E308 (NR_043572.1)	1390	99.50%	1564
POTR122	PV765703.1	*Virgibacillus olivae* E308 (NR_043572.1)	1411	99.36%	1564
POTR181	PV765705.1	*Virgibacillus olivae* E308 (NR_043572.1)	1385	99.49%	1564
POTR183	PV765707.1	*Virgibacillus marismortui* 123 (NR_028873.1)	1361	99.63%	1564
POTR191	PV802758.1	*Virgibacillus salarius* SA-Vb1 (NR_041270.1)	1399	99.63%	1564
